# The Regulatory Functions and the Mechanisms of Long Non-Coding RNAs in Cervical Cancer

**DOI:** 10.3390/cells11071149

**Published:** 2022-03-29

**Authors:** Qiwei Yang, Ayman Al-Hendy

**Affiliations:** Department of Obstetrics and Gynecology, University of Chicago, Chicago, IL 60637, USA; aalhendy@bsd.uchicago.edu

**Keywords:** cervical cancer, lncRNAs, gene expression, chromatin architecture, mRNA stability, sponge for miRNAs, ceRNA, epitranscriptomics, signaling pathways, p53

## Abstract

Cervical cancer is one of the leading causes of death in gynecology cancer worldwide. High-risk human papillomaviruses (HPVs) are the major etiological agents for cervical cancer. Still, other factors also contribute to cervical cancer development because these cancers commonly arise decades after initial exposure to HPV. So far, the molecular mechanisms underlying the pathogenesis of cervical cancer are still quite limited, and a knowledge gap needs to be filled to help develop novel strategies that will ultimately facilitate the development of therapies and improve cervical cancer patient outcomes. Long non-coding RNAs (lncRNAs) have been increasingly shown to be involved in gene regulation, and the relevant role of lncRNAs in cervical cancer has recently been investigated. In this review, we summarize the recent progress in ascertaining the biological functions of lncRNAs in cervical cancer from the perspective of cervical cancer proliferation, invasion, and metastasis. In addition, we provide the current state of knowledge by discussing the molecular mechanisms underlying the regulation and emerging role of lncRNAs in the pathogenesis of cervical cancer. Comprehensive and deeper insights into lncRNA-mediated alterations and interactions in cellular events will help develop novel strategies to treat patients with cervical cancer.

## 1. Introduction

Cervical cancer is a malignant neoplasm that arises from the uterine cervix cells. The cervix is made of two parts and is covered with two different types of cells. The endocervix is the part closer to the uterus and is made up of glandular cells, and the exocervix is the part next to the vagina, made up of squamous cells. The region where these two cell types converge, the transformation zone, is the region where most cervical cancers originate. Based on their origins, cervical cancers can be classified as squamous cell carcinomas, arising from the exocervical squamous cells and corresponding to about 80% of cervical cancer cases, adenocarcinomas, arising from the glandular cells of the endocervix and contributing to about 10–20% of cases, and adenosquamous carcinomas, wherein cancer possesses the features of both of the aforementioned cell types and is reported in a rare proportion of cases [1,2,3].

With an incidence of more than 600,000 and mortality of over 340,000 in 2020 [4], cervical cancer currently ranks as the fourth most common cancer worldwide, in both incidence and mortality. Despite the progress in diagnosis and therapeutic strategies, cervical cancer remains a leading cause of cancer deaths [5]. Human papillomavirus (HPV) is considered the major contributor to cervical carcinoma. Two oncogenic viral proteins, E6 and E7, can orchestrate diverse molecular mechanisms that may result in malignant cervical cancer progression. E6- and E7-induced altered transcriptional regulation resulted in genomic instability and distinguished the process of cell transformation from a productive viral infection and provided subsequent important steps towards malignancy [6]. For example, the constitutive expression of E6/E7 immortalizes primary epithelial cells and promotes tumor formation in vivo. E7 interacts with and stabilizes the retinoblastoma tumor suppressor family (RB1 and RB2), facilitating cell cycle transition from G1 to S phase. Similar to cell growth dysregulation, E6 interacting with ubiquitin ligase E6AP promotes tumor suppressor P53 degradation and cell proliferation [7,8]. In addition, other risk factors, including smoking, a weakened immune system, and chlamydia infection, also contribute to cervical cancer progression and carcinogenesis. To date, surgery (hysterectomy and trachelectomy), radiation therapy (external beam radiation and brachytherapy), and chemotherapy (cisplatin, cisplatin plus 5-fluorouracil (5-FU), carboplatin, paclitaxel, and topotecan) are the three main options for treatment of cervical cancer [5,9]. However, these clinical applications often have serious toxicity and side effects leading to their abrogation, resulting in the development of resistance by cancer cells. Moreover, about 80% of cervical cancer cases are reported at advanced stages, contributing to the poor prognosis and high mortality rates. Therefore, novel targeted therapeutic strategies are urgently needed to improve clinical outcomes.

## 2. Long Non-Coding RNAs and Potential Therapeutic Targeting in Cancer

Non-coding RNA (ncRNA) is commonly employed by RNA that does not encode a protein. These ncRNAs include microRNA, snoRNAs, other small regulatory RNAs, and lncRNAs [10,11,12]. The latter is a class of ncRNAs typically longer than 200 nucleotides. LncRNAs are mainly transcribed by RNA polymerase II, typically by a 5′7-methylguanosine cap and a 3′ poly (A) tail. Similar to messenger RNAs, they are involved in the pathogenesis of cancer [11,13,14,15,16,17,18]. Different classes of lncRNAs are generated from introns, exons, intergenic regions, telomerases, enhancers, or promoters [19]. Of the approximately 60,000 lncRNAs identified in human tumor tissues and cancer cell lines, the role and regulatory mechanisms of the majority of lncRNAs are still largely unknown. Nonetheless, the functional roles of numerous lncRNAs, whose expression is often dysregulated in various cancers, have been investigated. Accumulated studies have demonstrated that lncRNAs can regulate gene expression networks via the control of chromatin architecture and transcription in the nucleus, as well as via the modulation of mRNA stability, together with translation and post-translational modifications in the cytoplasm [20,21]. Furthermore, their functions and mechanisms are related to their genomic and intracellular localization. lncRNAs display functional similarity to typical protein-coding oncogenes and tumor suppressors involved in tumor initiation, progression, and metastasis. For example, HOX antisense intergenic RNA (HOTAIR) is one of the most well-studied oncogenic lncRNAs involved in the carcinogenesis of several types of cancer. An elevated HOTAIR expression is associated with resistance to chemotherapeutics, suggesting that inhibitors of HOTAIR could potentially resensitize a patient’s tumor to a specific chemotherapy [19,22,23,24]. One of the first-identified lncRNAs, H19, acts as a decoy for several tumor-suppressor miRNAs [25]. MEG3 lncRNA may serve as a tumor suppressor, and its downregulation has been associated with the development of a variety of human cancers [26].

LncRNAs are involved in a wide range of biological processes, including immune responses, a variety of disorders such as neuronal disorders, and cellular fate programs in cancer stem cells [27]. The latter plays a vital role in the origin and progression of malignancy and therapy resistance [28,29,30,31]. The regulatory network of lncRNAs includes EMT, drug resistance, and others via multiple mechanisms. LncRNAs have been shown to act as competing endogenous RNAs (ceRNAs) for specific microRNAs, thus regulating the expression of their downstream target genes [28]. LncRNAs promote cancer stem cells stemness and drug resistance [32]. In addition, lncRNAs regulate cell reprogramming, altering the transcriptome [33].

Detection of lncRNAs in body fluids (i.e., blood, saliva, urine, etc.) was also considered as a potential biomarker for the diagnosis, prognosis, and monitoring of the disease progression, as they acted as novel and potential drug targets for therapeutic options in carcinogenesis [34]. Furthermore, using body fluids to detect circulating lncRNAs is much less invasive when compared to collecting biopsies.

## 3. LncRNAs-Regulated Pathways in Cervical Cancer

Aberrant expression of lncRNAs has been previously and extensively reported in cervical cancer [35]. However, current studies (since March 2021) provide more progress about the importance of lncRNA dysregulation in promoting cervical cancer development, invasion, and metastasis through their interactions with several signaling pathways.

### 3.1. The Wnt Signaling Pathway

The Wnt signaling pathway is involved in various cellular processes such as embryogenesis, tissue renewal, cell proliferation, differentiation, and tumorigenesis. In canonical Wnt on signaling, Wnt binds to and activates the seven-pass transmembrane Frizzled (Fzd) receptor and the activated Fzd receptor recruits Dishevelled (Dvl) protein and AXIN. This blocks the formation of an AXIN-APC (adenomatous polyposis coli) complex and inhibits GSK3β. As a result, β-catenin avoids destruction in the cytoplasm and translocates into the nucleus. Subsequently, the nuclear β-catenin binds to the TCF/LEF transcription factors and triggers a β-catenin-regulated gene expression. In canonical Wnt off signaling, a combination of AXIN and APC allows GSK3β to phosphorylate β-catenin and targets it for proteasomal degradation [36].

**Figure 1:**
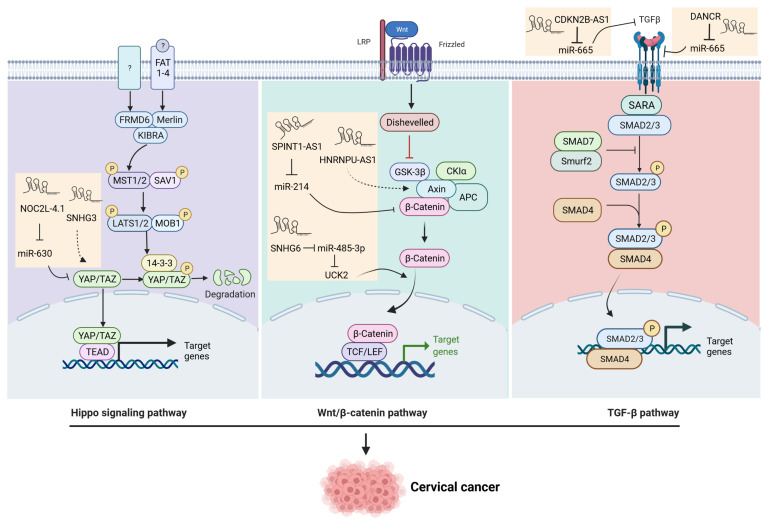
Schematic representation of Wnt, TGF-β, and Hippo pathways regulated by lncRNAs in cervical cancer.

Accumulated evidence demonstrates that LncRNAs play an essential role in the development and progression of a variety of cancers via the Wnt signaling pathway. For example, in cervical cancer, several lncRNAs, including *RP11-480112.5*, *ASB16-AS1*, *HOTAIR*, *CASC11*, *CALML3-AS1*, and *DANCER*, are involved in modulating Wnt Signaling pathways [35]. More recently, several additional lncRNAs, including SNHG6 [37], EGFR-AS1 [38], SPINT1-AS1 [39], HNRNPU-AS1 [40], and LINC00665 [41] have been identified that contribute to the proliferation, migration, invasion, and EMT in cervical cancer via the Wnt signaling pathway (Table 1, Figure 1).

### 3.2. The Mitogen-Activated Protein Kinase (MAPK) Pathway

The MAPK pathway plays a pivotal role in many cellular events, including cell migration, growth, apoptosis, and differentiation. The central components of this signaling pathway are ERK1/2, c-JNK N-terminal kinase, p38 MAPK, and ERK5, and after cascade phosphorylation, they are transmitted to the nucleus and regulate the expression of downstream targets [42]. Among them, JNK and p38 MAPK are activated by chemical, physical, and biological stimuli, while ERK1/2 are activated by cell growth factors. Tyrosine kinase receptors are the main receptors involved in the regulation of the MAPK signaling pathway. After receptor activation, RAS recruits RAF to phosphorylate MAPK, which subsequently activates ERK1/2. ERK1/2 in the nucleus activates several transcription factors such as MNK1, Elk-1, and c-Ets1. The MAPK pathway is upregulated in a variety of cancers. MAPK affects the secretion of extra growth factors and cytokines, leading to EMT progression.

Several lncRNAs are involved in the MAPK pathway contributing to cervical cancer progression. For example, over-expression of lncRNA CASC2 inhibits cell proliferation and migration by negatively regulating the MAPK pathway. In addition, TDRG1 activates MAPK1 by sponging miR-326, and TUG1 regulates cervical cancer sensitivity to cisplatin via the MAPK pathway. More recently, a study [43] showed that LOXL1-AS1 bound to miR-423-5p, and miR-423-5p targeted ENC1, which served as a regulator of the transcription factor Nrf2 played a key role in malignant transformation. A further study demonstrated that ENC1 knockdown decreased the protein levels of p-p38, p-MEK1/2, and p-ERK1/2, inhibiting the activation of the ERK/MEK pathway and reducing cell proliferation, suggesting that the LOXL1-AS1/miR-423-5p/ENC1 axis accelerates cervical cancer development through the MEK/ERK pathway. In addition, lncRNA LINC00997 was shown to activate the MAPK pathway-associated protein CUL2 by interacting with miR574-3p, demonstrating that the LINC00997/miR-574-3p/CUL2 axis contributes to proliferation, migration, invasion, and autophagy by activation of MAPK signaling in cervical cancer [44].

### 3.3. The TGF-β Signaling Pathway

TGF-β directs association with receptors on the plasma membrane, initiating the cascade of signal transduction that elicits biological actions on responding cells. The central mechanism of signal transduction by the TGF-β family receptors follows a well-characterized process of interactions and receptor-mediated phosphorylation. During the first step of TGF-β signaling, the TGF-β ligand binds to a heteromeric complex of type II and type I receptors. Upon ligand binding, the type II receptor phosphorylates and activates the type I receptor. The activated type I receptor, in turn, phosphorylates and activates the receptor-activated SMADs (R-SMADs), SMAD2, and SMAD3. SMAD7 competes with R-SMADs for interacting with type I receptors, thus preventing R-SMAD activation and proper propagation of the signaling. Activated R-SMADs dissociate from the type I receptors to form a complex with the common mediator SMAD4. Then, the trimeric complex (SMAD2, 3, 4) translocates into the nucleus, where it associates with high-affinity DNA binding transcription factors and chromatin remodeling proteins, therefore positively or negatively regulating the transcription of the TGF-β-responsive genes [45].

LncRNAs have been shown to be associated with the TGF-β pathway in various cancers [46]. Several studies have demonstrated that lncRNAs regulate TGF-β signaling, promoting cervical cancer progression. LncRNA DANCR regulates miR-665, which targets TGFBR1 through the ERK/SMAD pathway [47]. CDKN2B-AS1 is upregulated in cervical cancer tissues and cell lines and directly interacts with miR-181a-5p. TGF-β1 is a target of miR-181a-5p, suggesting that the CDKN2B-AS1/miR-181a-5p/TGF-β1 axis might play a vital role in cervical cancer development. LncRNA NEF suppresses HPV-negative cervical squamous cell carcinoma’s migration and invasion by inhibiting the TGF-β pathway. lncRNA loc285194 expression was downregulated in tissue samples and plasma from cervical squamous cell carcinoma patients. Plasma levels of loc285194 and TGF-ß1 significantly correlated with the presence of cervical squamous cell carcinoma. Moreover, lncRNA loc285194 overexpression downregulated TGF-β expression and resulted in a decrease in the cell migration of cervical cancer cells [48] (Figure 1).

### 3.4. The Hippo Signaling Pathway

The hippo signaling pathway is highly conserved and plays a critical role in tumorigenesis. It is characterized by phosphorylation of YAP1 and TAZ. Several factors can regulate the localization of YAP, which controls cell proliferation and apoptosis. Upon activation, MST1/2 kinase is phosphorylated and forms a complex with SAV1 to phosphorylate LATS1/2, which in turn phosphorylates the transcriptional co-activator YAP. The phosphorated YAP binds to the 14-3-3 proteins and is retained in the cytoplasm or is degraded by the ubiquitin-proteasome pathway [49]. In the absence of an active Hippo signaling pathway, unphosphorylated Yap and Taz enter the nucleus, interact with the transcription factors, and stimulate the expression of genes involved in proliferation and anti-apoptotic processes. In addition, the Hippo pathway also plays a critical role in stem cell and tissue-specific progenitor cell self-renewal and expansion.

Accumulated evidence demonstrates that lncRNAs can promote the oncogenic signaling of YAP in a variety of cancers [50,51,52,53,54]. In cervical cancer, several studies showed that lncRNAs regulated the Hippo pathway contributing to the progression of cancer. The expression of lncRNA NOC2L-4.1 was upregulated in cervical cancer, and the downregulation of NOC2L-4.1 suppressed cell migration and proliferation. Further studies revealed the critical role of the NOC2L-4.1/miR-630/YAP regulatory network in promoting cervical cancer progression [55]. Another LncRNA, SNHG3, is involved in the occurrence and development of various cancers. In cervical cancer, SNHG3 promotes the proliferation, migration, and invasion of cervical cancer cells in vitro, and facilitates cervical cancer growth in vivo. Notably, SHHG3 interacted with YAP1, thus inhibiting its degradation, concomitantly with altered expression of several YAP1 target genes [56] (Figure 1).

### 3.5. DNA Damage Repair (DDR) and Genomic Integrity

The DNA damage response (DDR) pathway is a complex regulatory network responsible for identifying disruptions in DNA structure, integrity, and stability. DDR is an evolutionarily conserved process that maintains genomic integrity but is frequently dysregulated in cancer. Damaged DNA bases and DNA single-strand breaks are the most abundant types of DNA damage. Although DNA double-strand breaks are less common, they are considered the most deleterious types of DNA damage [57]. Although this system normally protects healthy cells from tumorigenic DNA damage and replication errors, most cancer cells acquire some form of enhanced DDR that eventually results in radiotherapeutic or chemotherapeutic resistance. The function and capacity of DDR machinery are essential to ensure the maintenance of normal cycling cells and prevent the accumulation of mutations that increase the potential for malignancy. Recently, several studies demonstrated that DDR is associated with the dysregulation of lncRNAs that are implicated in cancer progression [58]. lncRNA LINP1 was upregulated in cervical cancers compared to adjacent tissues. LINP is associated with the non-homologous end joining (NHEJ) pathway proteins Ku80 and DNA-PKcs in cervical cancer cell lines by RNA pull-down assay. Knockdown of LINP1 increased irradiation-induced cell apoptosis and delayed the repair of DNA double-strand breaks [59]. Another lncRNA, LINC02535, cooperated with PCBP2 and regulated RRM1 mRNA stability to accelerate cell proliferation and EMT by facilitating the repair of DNA damage in cervical cancer cells [60].

### 3.6. The Phosphatidylinositol 3- kinase/Protein Kinase B (PI3K/AKT) Pathway

PI3K is a member of the lipid kinases family. In the normal state of the cell, various extracellular factors, such as hormones, growth factors, and cytokines, send signals to activate PI3K through an interaction with a phosphorylated tyrosine receptor. A PI3K downstream cascade generates signals received by its targets, the most important one being the protein kinase B (AKT) that dominates the signal transduction of the PI3K pathway [61]. Activation of AKT is a common phenomenon in human cancers, leading to the promotion of cell proliferation [62]. The entire PI3K/AKT signaling pathway regulates the cell physiology and pathology, including apoptosis, cell proliferation, invasion, and metastasis [62,63,64]. This pathway is abnormally activated in different tumors, including cervical cancer [65,66].

Several lncRNAs regulate the PI3K/AKT pathways in cervical cancer. Decreased expression of lncRNA ANRIL suppressed cell proliferation, migration, and invasion when the PI3K/AKT pathway was inactivated, suggesting that ANRIL inhibits cervical cancer progression via the PI3K/AKT pathway. In addition, downregulating RP1-93H18.6 decreased cell proliferation and EMT, while promoting apoptosis by blocking the PI3K/AKT/mTOR signaling pathway [35].

More recently, several additional lncRNAs were found to be involved in cervical cancer progression via the PI3K pathway. Knockdown of lncRNA KCNQ1OT1 caused apoptosis by sponging miR-1270, thereby altering the expression of LOXL2. Moreover, decreased expression of KCNQ1OT1 reduced the p-AKT levels in cervical cancer cells [67]. LncRNA HOTAIR triggered the migration and proliferation of cervical cancer cells and promoted chemoresistance by facilitating EMT via the miR-29b/PTEN/PI3K axis [67]. LINC00861 functions as a ceRNA for miR-513b-5p to inhibit the progression of cervical cancer cells and modulate the PTN/AKT/mTOR signaling pathway [68]. Similarly, lncRNA LINC00673 exerts oncogenic function in cervical cancer through the PTEN/AKT pathway [69].

### 3.7. The Hypoxia Signaling Pathway

Hypoxia is an environmental stressor instigated by low oxygen availability and is one of the major factors that contribute to cancer progression and the acquisition of chemotherapeutic resistance in many ways. Hypoxia-inducible factors-1 and -2 alpha (HIF-1α and EPAS1/HIF-2α) function as master regulators of the adaptive response to hypoxia. HIF-induced genes promote characteristic tumor behaviors, including angiogenesis and metabolic reprogramming. Therefore, targeting the signaling pathways associated with hypoxia is deemed attractive for achieving tumor suppression, as well as for mitigating immunosuppression and improving therapeutic outcomes [70].

Accumulating evidence shows that lncRNAs are modulated by hypoxia during oncogenesis [71]. Several studies have demonstrated that lncRNAs are involved in the hypoxia pathway regulation in cervical cancer. SNHG15 is upregulated in cervical cancer tissues and promotes cervical cancer progression via the miR-4735-3p/HIF1a axis [72]. The expression of LncRNA OIP5-AS1 is increased in cervical cancer and correlates with unfavorable outcomes. For example, OIP5-AS1 expression in cervical cancer tissues is significantly related to tumor size, differentiation, lymph node metastasis, and FIGO stages of cervical cancer. In addition, the high levels of OIP5-AS1 correlate with poor 5-year overall survival. OIP5-AS1 is also a hypoxia-responsive lncRNA and is essential for hypoxia-enhanced glycolysis, which is dependent on IDH2 or hypoxia-inducible factor-1α (HIF-1α) [73]. TDRG1 promotes hypoxia-induced glycolysis via the miR-214-5p/SEMA4C axis in cervical cancer cells [74]. LncRNA ANCR downregulates hypoxia-inducible 1 alpha and suppresses the growth of HPV-negative cervical squamous carcinoma cells under hypoxic conditions [75].

### 3.8. The p53 Pathway

p53 is a nuclear transcription factor and transactivates numerous target genes involved in cell cycle arrest and apoptosis [76]. Under normal conditions, p53 is expressed at an extremely low level and is caused by proteasomal degradation mediated largely by the RING-finger type E3 ubiquitin protein ligase, MDM2, in a functionally latent form. Upon DNA damage, p53 accumulates in the cell nucleus through post-translational modifications such as phosphorylation and acetylation. These chemical modifications convert p53 from a latent to an active form, which might be due to the dissociation of MDM2 from p53. The activation of the p53 protein as a transcription factor initiates several cellular events, including a program of cell cycle arrest, cellular senescence, or apoptosis. The transcriptional network of p53-responsive genes produces proteins that interact with many other signal transduction pathways in the cell, and a number of positive and negative autoregulatory feedback loops act upon the p53 response [77].

The link between lncRNAs and the p53 pathway has been investigated in several types of cancer [78,79,80,81,82,83,84]. Notably, p53-related lncRNAs in cervical cancer have also been reported. For example, lncRNA RPL34-AS1 induces RPL34, therefore inhibiting cervical cancer cell proliferation, invasion, and metastasis through modulation of the MDM2-P53 signaling pathway [85]. LncRNA DINO is a p53 transcriptional target that has been reported to bind to and stabilize p53, thereby amplifying p53 signaling. In cervical cancer, DINO is downregulated, and upregulation of DINO causes p53 reactivation in HPV-positive cervical cancer cells [86]. lncRNA WT1-AS suppresses cell proliferation, migration, and invasion via the miR-330-5p/p53 axis in cervical cancer [87]. In addition to lncRNAs that regulate p53, a number of p53-induced lncRNAs, including *PINCR*, *PINT*, *PURPL*, *LincRNA-p21*, *TUG1*, *NEAT*, and DINO, among others, have been identified and reported in several diseases [84,88,89,90,91,92,93,94,95,96]. These p53-induced lncRNAs play a critical role in multiple cellular processes, including cell proliferation and apoptosis. Moreover, reciprocal modulation of lncRNAs and p53 has been shown to regulate tumorigenesis [91]. Importantly, p53 is wild-type or non-mutated in most cervical cancer patients. Therefore, the identification of p53-induced lncRNAs in cervical cancer will help better understand the role and regulatory mechanism of the p53 pathway in cervical cancer.

### 3.9. Other lncRNA-Related Targets in Cervical Cancer

The high-throughput approach has been used to determine the differentially expressed lncRNAs in cervical cancer extensively and has identified over 3000 differentially expressed lncRNAs, including sense, antisense, intronic, intergenic, and bidirectional lncRNAs, in cervical cancer tissues compared with adjacent non-cancerous tissues. These lncRNAs with different expression patterns may play important roles in the development and progression of cervical cancer [97]. Another experiment was performed using a high-throughput RNA sequencing approach and identified 19 differentially expressed lncRNAs in HPV16-mediated cervical squamous cell carcinoma and matched adjacent non-tumor tissues. Among them, 11 lncRNAs participated in at least one pathway related to cancer, and some lncRNAs may also be involved in the immune system, signal transduction, and cellular community [98]. lnc_000231, as one of the 19 identified differentially expressed lncRNAs, was further investigated, and it was demonstrated that HPV oncoprotein E6 increased lnc_000231 expression by triggering H3K4me3 modification in the lnc_000231 promoter region by destabilizing histone demethylase KDM5C. Furthermore, a functional analysis demonstrated that lnc_000231 promoted cervical cell proliferation and tumor formation. Notably, a series of studies, including ChIP-PCR, site-directed mutation, knockdown, and promoter activity experiments, revealed the close link between lnc_000231 and miR-497-5p [7].

LncRNAs can indirectly regulate RNA expression by sequestering miRNAs and acting as ceRNAs or as sponges [99], therefore lncRNAs decrease miRNAs regulatory effect on mRNAs and introduce an additional layer of complexity in the miRNA-related network. Recently, lncRNA-miRNA interactions have been investigated in cervical cancer (Table 1). For example, LncRNA MAGI2-AS3 suppresses the proliferation and invasion of cervical cancer cells by regulating the miR-15b/CCNE1 axis. LncRNA ZFAS1 (Zinc finger antisense 1) impacts cervical cancer growth through miR-190a-3p. KLF6 is negatively regulated by MiR-190a-3p, but positively regulated by ZFAS1. Inhibition of ZFAS1 reduces cervical cancer tumor growth and the expression levels of KLF6 but increases the expression levels of miR-190a-3p. Therefore, ZFAS1 could regulate cervical cancer pathogenesis by regulating the miR-190a-3p/KLF6 axis [100]. LncRNA 885 (LINC00885) exerts oncogenic function in cervical cancer by regulating the miR-3150b-3p/BAZ2A axis [101]. Several lncRNAs are involved in cell proliferation, apoptosis, and cell cycle correlating to abnormal cell proliferation of cervical cancer cells. For example, OTUD6B-AS1 targets cyclinD2 via miR-206. LINC00313 alters CDK6 via sponging miR-4677-3p. FOXD2-AS1 promotes cervical cancer progression by decreasing the p21 transcription [102] (Table 1). In addition, one lncRNA can interact with many miRNAs via the sponge mechanism and therefore target multiple mRNAs and signalings in cervical cancer. For example, HOTAIR interacted with miR-29b, miR-203, and miR-214-3p, and targeted PI3K and Wnt pathways in cervical cancer (Table 1). FEZF1-AS interacted with miR-1254 and miR-367-3p involved in the proliferation, migration, and invasion of cervical cancer (Table 1). NEAT1 sponged miR-377 and miR-34a, and targeted FGFR1 and LDHA, respectively. KCNQ1OT1 interacted with MiR-1270 and miR-296-5p and altered the expression of LOXL2 and HYOU1, respectively (Table 1). Identified lncRNAs that interacted with miRNAs in cervical cancer are summarized in Table 1.

**Table 1:** Role and mechanisms of lncRNAs in cervical cancer (Papers published Since March 2021).

LncRNA	Biological Samples	Interaction with RNA	Targets/Pathway	Biological Processes	Publication Time	Ref.
AC010198.2	Cells	miR-34b-3p	STC2	Drug resistance	Oct. 2021	[103]
AFAP1-AS1	Cells	miR-27b-3p	VEGF-C	stemness	July 2021	[104]
AL592284.1	Cells and tissues	miR-30a-5p	Vimentin/EMT	proliferation, metastasis	Nov. 2021	[105]
ALOX12-AS1	Tissues	miR-3171	NA	proliferation	Jan. 2022	[106]
ANXA2P2	Cells and tissues	miR-361-3p	SOX9	cisplatin-resistant	Jan. 2022	[107]
CASC9-1	Cells	miR-383-5p	MAPKAP1	proliferation, migration, invasion, apoptosis	Nov. 2021	[108]
CCAT2	Cells and tissues	miR-493-5p	CREB1	proliferation, EMT, tumor growth	Dec. 2021	[109]
DANCR	Cells and tissues	miR-145-3p	ZEB1	tumor growth, metastasis	July 2021	[110]
DARS-AS1	Cells and tissues	NA	ATP1B2, cGMP-PKG	proliferation, invasion, migration	June 2021	[111]
DLEU2	Cells	NA	ZFP36, p53, notch signaling, p53	proliferation, cell cycle	May 2021	[112]
DUXAP8	Cells	miR-1297	RCN2	malignancy, tumor growth	Nov. 2021	[113]
EGFR-AS1	Cells	miR-2355-5p	ACTN4-mediated Wnt	proliferation, migration, invasion, apoptosis	Jan. 2022	[38]
FBX19-AS1	Cells	miR-193a-5p	COL1A1	proliferation, migration, invasion, EMT, apoptosis, metastasis	Aug. 2021	[114]
FEZF1-AS1	Cells and tissues	miR-1254	NA	proliferation, migration, invasion	July 2021	[115]
FEZF1-AS1	Cells	miR-367-3p	SLS12AS	proliferation, migration, invasion, apoptosis	Jan. 2022	[115]
FGD5-AS1	Cells	miR-129-5p	BST2	macrophage M1 polarization	Oct. 2021	[116]
FOXD2-AS1	Cells and tissues	NA	METTL3, LSD1/p21	proliferation, migration, tumor growth	July 2021	[102]
FOXD3-AS1	Cells and tissues	miR-128-3p	LIMK1	proliferation, migration, invasion	May 2021	[117]
HAND2-AS1	Tissues	miR-21-5p	TIMP3/VEGFA	proliferation, migration, invasion, apoptosis, tumor growth	June 2021	[118]
HNRNPU-AS1	Cells and tissues	miR-205-5p	AXIN2, Wnt	proliferation, apoptosis, tumor growth	Sep. 2021	[40]
HOTAIR	Cells and tissues	NA	Wnt	drug resistance	Oct. 2021	[119]
HOTAIR	Cells	miR-29b	PTEN/PI3K	proliferation, drug resistance, EMT	Sep. 2021	[120]
HOTAIR	Stem cells	miR-203	ZEB1	EMT	Sep. 2021	[121]
HOTAIR	Cells	miR-214-3p	NA	proliferation, apoptosis	July 2021	[122]
HOTAIR HOXA-AS2	Cells and tissues	miR-509-3p	BTN3A1	proliferation, migration, invasion, tumor growth	Sep. 2021	[123]
HOXC13-AS	Cells, and tissues	NA	FTO, Wnt, FZD	proliferation, invasion, EMT	July 2021	[124]
HOXC-AS3	Cells and tissues	miR-105-5p	SOS1	proliferation, migration, invasion, apoptosis	Oct. 2021	[125]
HOXD-AS1	Cells	NA	FRRS1	proliferation, apoptosis, tumor growth	Jan. 2022	[126]
ILF3-AS1	Cells and tissues	miR-454-3p	PTEN	survival rate, migration, apoptosis, EMT	Aug. 2021	[127]
KCNQ1OT1	Cells	miR-1270	LOXL2, PI3K/Akt	viability, apoptosis	Feb. 2022	[67]
KCNQ1OT1	Cells and tissues	miR-296-5p	HYOU1	proliferation, migration, invasion, tumor growth	Dec. 2021	[128]
LIN01006	Cells and tissues	miR-28-5p	PAK2	proliferation, migration, invasion, tumor growth	April 2021	[129]
LINC00313	Cells and tissues	miR-4677-3p	CDK6	migration, EMT	Mar. 2021	[130]
LINC00514	Cells and tissues	miR-708-5p	HOXB3	Proliferation, invasion	Jan. 2022	[131]
LINC00662	Cells	miR-103a	PDK4	proliferation, apoptosis	June 2021	[132]
LINC00665	Cells	NA	CTNNB1, Wnt signaling	proliferation, migration, invasion, EMT	April 2021	[41]
LINC00673	Cells and serum	NA	cell cycle, p53 pathway	proliferation, cell cycle, tumor growth	May 2021	[133]
LINC00707	Cells and tissues	miR-382-5p	VEGFA	proliferation, tumor growth	June 2021	[134]
LINC00885	Cells and tissues	miR-3150b-3p	BAZ2A	Proliferation, apoptosis, tumor growth	Jan. 2022	[101]
LINC00885	Cells and tissues	NA	NA	proliferation, invasion, EMT	June 2021	[135]
LINC00899	Cells and tissues	miR-944	ESR1	proliferation, migration, invasion	June 2021	[136]
LINC00997	Cells	miR-574-3p	CUL2, MAPK	proliferation, migration, invasion, autophagy	July 2021	[44]
LINC01133	Cells and tissues	miR-30a-5p	FOXD1	proliferation, metastasis, apoptosis	June 2021	[137]
LNMAS	Tissues	NA	TWIST, STC1	metastasis, EMT, immune evasion	Feb. 2022	[138]
LOXL1-AS1	Cells and tissues	miR-423-5p	ENC1, MEK/ERK pathway	proliferation, migration, invasion, angiogenesis, tumor growth	Jan. 2022	[43]
MAG12-AS3	Tissues	miR-15b	CCNE1	proliferation, invasion	Jan. 2022	[139]
MALAT1	Cells and tissues	NA	NA	proliferation, invasion, migration	Aug. 2021	[140]
MALAT1	Cells	miR-485-5p	MAT2A	proliferation	Nov. 2021	[141]
MALAT1	Cells and tissues	miR-124-5p	NA	proliferation, tumor growth	Nov. 2021	[142]
MEF2C-AS1	Cells and tissues	miR-592	RSPO1	proliferation, migration, invasion	June 2021	[143]
MEG3	Cells and tissues	miR-7-5p	STC1	ERs-mediated apoptosis	May 2021	[144]
MiIR503HG	Cells and tissues	miR-191	CEBPB	proliferation, metastasis, apoptosis	April 2021	[145]
NEAT1	Cells and tissues	miR-377	FGFR1	proliferation, migration, apoptosis	Jan. 2022	[146]
NEAT1	Cells and tissues	miR-34a	LDHA	drug-resistant, glycolysis rate	July 2021	[147]
OIP5-AS1	Cells and tissues	miR-124-5p	IDH2/HIF1a	proliferation, Hypoxia, Warburg effect	Aug. 2021	[73]
OIP5-AS1	Cells and tissues	MiR-147a	IGF1R, E-cadherin	migration, invasion, EMT	Mar. 2022	[148]
OTUD6B-AS1	Cells	miR-206	cyclinD2	drug-resistant	Oct. 2021	[149]
RPL34-AS1	Cells	NA	RPL34	proliferation, migration, invasion,	May 2021	[85]
SNHG15	Cells and tissues	miR-4735-39	HIF1a	tumor progression	Jan. 2022	[72]
SNHG17	Serum	miR-375-3p	NA	proliferation, migration, invasion	June 2021	[150]
SNHG3	Cells and tissues	NA	YAP1	proliferation, migration, invasion, tumor growth	Jan. 2022	[56]
SNHG5	Cells and tissues	miR-132	SOX4	proliferation, migration, invasion	Mar. 2021	[151]
SNHG6	Cells and tissues	miR-485-3p	UCK2, Wnt	proliferation, migration, invasion, EMT	Nov. 2021	[37]
SPINT1-AS1	Cells and tissues	miR-214	Wnt	proliferation, migration, invasion, tumor growth	July 2021	[39]
TDRG1	Cells and tissues	miR-214-5p	SEMA4C	invasion, tumor growth, hypoxia-induced glycolysis	April 2021	[74]
UCA1	Cells and tissues	miR-299-3p	NA	proliferation, invasion	Nov. 2021	[152]
UNC5B-AS1	Cells and tissues	miR-4455	RSPO4	proliferation, migration, invasion, apoptosis	Dec. 2021	[153]
USP30-AS1	Cells and tissues	miR-299-3p	PTP4A1	proliferation, migration, invasion, tumor growth	July 2021	[154]
WT1-AS	Cells and tissues	NA	SPL1/PIK3AP1	proliferation, autophagy, apoptosis, tumor progression	July 2021	[155]
WTA-AS	Cells and tissues	miR-205	NA	cell cycle, apoptosis, migration, invasion, EMT, tumor growth	Dec. 2021	[156]
ZFAS1	Cells	MiR-190a-3p	KLF6	proliferation, invasion, migration	Feb. 2022	[100]

Note: NA: not available.

## 4. Epitranscriptomic Regulation of lncRNAs in Cervical Cancer

The epitranscriptome refers to the complete ensemble of chemical modifications affecting the RNA transcripts (coding and non-coding RNAs), and epitranscriptomics is an emerging field in molecular medicine with vast potential. Epitranscriptomics research encompasses many RNA modifications (more than 160 types of modifications) without changes in their sequences, which are widespread among RNA transcripts. Three kinds of key proteins manage the tunable modifications of RNA between generations: writers, erasers, and readers [157,158,159,160]. In the lncRNA transcriptome, N^6^-methyladenosine (m^6^A) contains the highest amount and a wide range of important functions, followed by m^5^C and ψ [161]. Elucidating the modification of lncRNAs provides a fundamental pathway for understanding gene regulation and relevant cellular processes that contribute to disease development.

m^6^A-modified lncRNAs were reported to serve as potential biomarkers for predicting prognoses and immune response in patients with cervical cancer [162]. Several lncRNAs have been regulated via an epitranscriptomic mechanism. lncRNA FOXD2-ASZ1 expression was significantly upregulated in cervical cancer cells and tissues, correlating with the unfavorable prognosis. The functional assays demonstrated that FOXD2-AS1 promoted migration and proliferation of cervical cancer cells, while FOXD2-AS1 silencing repressed the tumor growth in vivo. Notably, m^6^A “writer”, METTL3, enhanced the stability of FOXD2-AS1 and recruited lysine-specific demethylase 1 (LSD1) to the promoter region of p21 to silence its transcription abundance [102]. Dysregulation of another lncRNA KCNMB2-AS1 correlated with poor cervical cancer outcomes. The higher KCNMB2-AS1 expression associated with a shorter survival time. Mechanically, KCNMB2-AS1 was predominantly located in the cytoplasm and served as a ceRNA to abundantly sponge miR-130b-5p and miR-4294, resulting in the upregulation of IGF2BP3, a well-documented oncogene in cervical cancer. Moreover, IGF2BP3 acted as an m^6^A “reader” to bind m^6^A-modified KCNMB2-AS1, therefore stabilizing KCNMB2-AS1. Thus, KCNMB2-AS1 and IGF2BP3 formed a positive regulatory circuit that enlarged the tumorigenic effect of KCNMB2-AS1 in cervical cancer [163]. In addition, the lncRNA GAS5-AS1 expression in cervical cancer tissues was markedly decreased compared to adjacent normal tissues. The downregulation of GAS5-AS1 significantly correlated with poor outcomes in patients with cervical cancer. The Kaplan-Meier survival curve and the log-rank test demonstrated that patients with high-level GAS5-AS1 had better overall survival than those with low-level GAS5-AS1. GAS5-AS1 drastically reduced cell proliferation, migration, and invasion in vitro. It also remarkably suppressed tumorigenicity and metastasis in vivo via interacting with the tumor suppressor GAS5 and increased its stability by interacting with RNA demethylase ALKBH5 and decreasing m^6^A modification of GAS5. Moreover, m^6^A-mediated GAS5 RNA degradation was shown to rely on the m^6^A reader protein YTHDF2-dependent pathway [164]. LncRNA HOXC13-AS is also regulated via the m^6^A mechanism. HOXC13-AS was elevated in cervical cancer and positively correlated with FZD6, a ‘Frizzled’ gene family member serving as a receptor for Wnt signaling proteins. FTO acted as an m^6^A “eraser” to reduce m^6^A and stabilize HOXC13-AS linking to the upregulation of HOXC13-AS in cervical cancer [124].

## 5. Conclusions and Future Perspectives

**Figure 2:**
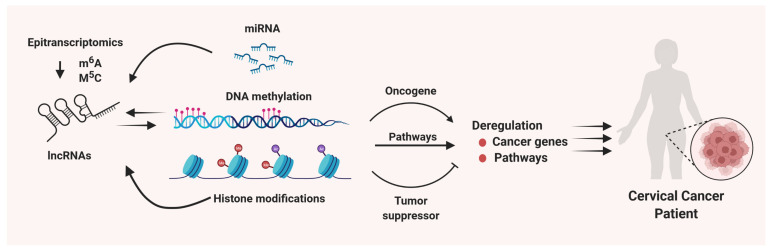
Role and regulatory mechanism of lncRNAs in cervical carcinogenesis. lncRNAs can regulate gene expression networks via regulating microRNAs, histone modifications, DNA methylation dynamics and others, resulting in altering the key pathways and oncogenes/tumor suppressor genes triggering the pathogenesis of cervical carcinogenesis. In addition, aberrant expression of lncRNAs in cervical cancer is modulated by epitranscriptional, epigenetic, and other mechanisms.

Considerable progress has been made in recent years to study the role and molecular mechanisms underlying the lncRNA-mediated cellular events which contribute to the initiation, development, and progression of cancers, including cervical cancer. LncRNAs can be regulated via epitranscriptomic and epigenetic mechanisms. LncRNAs can interact with epigenome and miRNAs resulting in altering the transcriptome and dysregulating the oncogenes and tumor suppressors, therefore leading to cervical cancer progression (Figure 2). However, several aspects need to be further elucidated, including (1) characterizing the interaction of lncRNAs with other RNAs, (2) elucidating the interaction of lncRNA with chromatin structure and other epigenetic regulations, (3) characterizing the role of lncRNAs in transcriptional regulation via interacting with DNA and proteins, and (4) investigating the role of lncRNAs in post-transcriptional regulation in cervical cancer. This deep mechanistic investigation will better understand the drug resistance, immune escape, and environmental impact in cervical cancer. Thorough research of lncRNAs is needed to characterize their functional role in cervical cancer. Clinical application of molecular inhibitors targeting unique lncRNAs might provide a novel option for the personalized treatment of cancer patients.

In conclusion, lncRNAs play a critical role in abnormal proliferation, migration, invasion, EMT, and tumor growth in cervical cancers via multiple mechanisms, which provide potential targets for treating female patients with cervical cancer. Comprehensive and deep insights into molecular mechanisms underlying the pathogenesis of cervical cancer will offer novel and better options for early detection, diagnosis, and treatment for this aggressive disease.

## References

[1] Ijff M., Crezee J., Oei A.L., Stalpers L.J., Westerveld H. (2022). The role of hyperthermia in the treatment of locally advanced cervical cancer: A comprehensive review. Int. J. Gynecol. Cancer.

[2] Muthusami S., Sabanayagam R., Periyasamy L., Muruganantham B., Park W.Y. (2021). A review on the role of epidermal growth factor signaling in the development, progression and treatment of cervical cancer. Int. J. Biol. Macromol..

[3] Regauer S., Reich O. (2021). The origin of Human Papillomavirus (HPV)—induced cervical squamous cancer. Curr. Opin. Virol..

[4] Sung H., Ferlay J., Siegel R.L., Laversanne M., Soerjomataram I., Jemal A., Bray F. (2021). Global Cancer Statistics 2020: GLOBOCAN Estimates of Incidence and Mortality Worldwide for 36 Cancers in 185 Countries. CA Cancer J. Clin..

[5] Ghosh S., Jayaram P., Kabekkodu S.P., Satyamoorthy K. (2022). Targeted drug delivery in cervical cancer: Current perspectives. Eur. J. Pharmacol..

[6] Snijders P.J.F., Steenbergen R., Heideman D.A.M., Meijer C.J.L.M. (2006). HPV-mediated cervical carcinogenesis: Concepts and clinical implications. J. Pathol..

[7] Zhang Y., Li X., Zhang J., Mao L. (2020). E6 hijacks KDM5C/lnc_000231/miR-497-5p/CCNE1 axis to promote cervical cancer progression. J. Cell Mol. Med..

[8] White E.A., Kramer R.E., Tan M.J.A., Hayes S.D., Harper J.W., Howley P.M. (2012). Comprehensive Analysis of Host Cellular Interactions with Human Papillomavirus E6 Proteins Identifies New E6 Binding Partners and Reflects Viral Diversity. J. Virol..

[9] Zheng J., Chen L. (2021). Non-coding RNAs-EZH2 regulatory mechanisms in cervical cancer: The current state of knowledge. Biomed. Pharmacother..

[10] Mattick J.S., Makunin I.V. (2006). Non-coding RNA. Hum. Mol. Genet..

[11] Feng T., Wu Q.F. (2022). A review of non-coding RNA related to NF-kappaB signaling pathway in the pathogenesis of osteoarthritis. Int. Immunopharmacol..

[12] Yang Q., Al-Hendy A. (2018). Non-coding RNAs: An important regulatory mechanism in pathogenesis of uterine fibroids. Fertil. Steril..

[13] Wu Z., Lu Z., Li L., Ma M., Long F., Wu R., Huang L., Chou J., Yang K., Zhang Y. (2022). Identification and Validation of Ferroptosis-Related LncRNA Signatures as a Novel Prognostic Model for Colon Cancer. Front. Immunol..

[14] Wang Z., Lin M., He L., Qi H., Shen J., Ying K. (2021). Exosomal lncRNA SCIRT/miR-665 Transferring Promotes Lung Cancer Cell Metastasis through the Inhibition of HEYL. J. Oncol..

[15] Wu B., Xue X., Lin S., Tan X., Shen G. (2022). LncRNA LINC00115 facilitates lung cancer progression through miR-607/ITGB1 pathway. Environ Toxicol..

[16] Wu Q.-N., Luo X.-J., Liu J., Lu Y.-X., Wang Y., Qi J., Liu Z.-X., Huang Q.-T., Lu J.-B., Jin Y. (2021). MYC-Activated LncRNA *MNX1-AS1* Promotes the Progression of Colorectal Cancer by Stabilizing YB1. Cancer Res..

[17] Zhang H., Wang J., Yin Y., Meng Q., Lyu Y. (2021). The role of EMT-related lncRNA in the process of triple-negative breast cancer metastasis. Biosci. Rep..

[18] Yang C., Chen K. (2022). Long Non-Coding RNA in Esophageal Cancer: A Review of Research Progress. Pathol. Oncol. Res..

[19] Di Fiore R., Suleiman S., Felix A., O’Toole S., O’Leary J., Ward M., Beirne J., Sabol M., Ozretić P., Yordanov A. (2021). An Overview of the Role of Long Non-Coding RNAs in Human Choriocarcinoma. Int. J. Mol. Sci..

[20] Yao R.-W., Wang Y., Chen L.-L. (2019). Cellular functions of long noncoding RNAs. Nat. Cell Biol..

[21] Zhang X., Wang W., Zhu W., Dong J., Cheng Y., Yin Z., Shen F. (2019). Mechanisms and Functions of Long Non-Coding RNAs at Multiple Regulatory Levels. Int. J. Mol. Sci..

[22] Niinuma T., Suzuki H., Nojima M., Nosho K., Yamamoto H., Takamaru H., Yamamoto E., Maruyama R., Nobuoka T., Miyazaki Y. (2012). Upregulation of miR-196a and HOTAIR Drive Malignant Character in Gastrointestinal Stromal Tumors. Cancer Res..

[23] Kogo R., Shimamura T., Mimori K., Kawahara K., Imoto S., Sudo T., Tanaka F., Shibata K., Suzuki A., Komune S. (2011). Long Noncoding RNA HOTAIR Regulates Polycomb-Dependent Chromatin Modification and Is Associated with Poor Prognosis in Colorectal Cancers. Cancer Res..

[24] Naz F., Tariq I., Ali S., Somaida A., Preis E., Bakowsky U. (2021). The Role of Long Non-Coding RNAs (lncRNAs) in Female Oriented Cancers. Cancers.

[25] Kallen A.N., Zhou X.-B., Xu J., Qiao C., Ma J., Yan L., Lu L., Liu C., Yi J.-S., Zhang H. (2013). The Imprinted H19 LncRNA Antagonizes Let-7 MicroRNAs. Mol. Cell.

[26] Sheng X., Li J., Yang L., Chen Z., Zhao Q., Tan L. (2014). Promoter hypermethylation influences the suppressive role of maternally expressed 3, a long non-coding RNA, in the development of epithelial ovarian cancer. Oncol. Rep..

[27] Statello L., Guo C.-J., Chen L.-L., Huarte M. (2021). Gene regulation by long non-coding RNAs and its biological functions. Nat. Rev. Mol. Cell Biol..

[28] Fan B., Zhang Q., Wang N., Wang G. (2022). LncRNAs, the Molecules Involved in Communications with Colorectal Cancer Stem Cells. Front. Oncol..

[29] McCabe E.M., Rasmussen T.P. (2020). lncRNA involvement in cancer stem cell function and epithelial-mesenchymal transitions. Semin. Cancer Biol..

[30] Wang X.-C., Liu Y., Long F.-W., Liu L.-R., Fan C.-W. (2021). Identification of a lncRNA prognostic signature-related to stem cell index and its significance in colorectal cancer. Futur. Oncol..

[31] Wu Y., Wang T., Xia L., Zhang M. (2021). LncRNA WDFY3-AS2 promotes cisplatin resistance and the cancer stem cell in ovarian cancer by regulating hsa-miR-139-5p/SDC4 axis. Cancer Cell Int..

[32] Eptaminitaki G.C., Wolff N., Stellas D., Sifakis K., Baritaki S. (2021). Long Non-Coding RNAs (lncRNAs) in Response and Resistance to Cancer Immunosurveillance and Immunotherapy. Cells.

[33] Kanwal S., Guo X., Ward C., Volpe G., Qin B., Esteban M.A., Bao X. (2020). Role of Long Non-coding RNAs in Reprogramming to Induced Pluripotency. Genom. Proteom. Bioinform..

[34] Wang W., Min L., Qiu X., Wu X., Liu C., Ma J., Zhang D., Zhu L. (2021). Biological Function of Long Non-coding RNA (LncRNA) Xist. Front. Cell Dev. Biol..

[35] Cáceres-Durán M.Á., Ribeiro-Dos-Santos Â., Vidal A.F. (2020). Roles and Mechanisms of the Long Noncoding RNAs in Cervical Cancer. Int. J. Mol. Sci..

[36] Hamidi A.A., Khalili-Tanha G., Navaei Z.N., Moghbeli M. (2022). Long non-coding RNAs as the critical regulators of epithelial mesenchymal transition in colorectal tumor cells: An overview. Cancer Cell Int..

[37] Wei J., Gao Y., Li Z., Jia H., Han B. (2021). LncRNA SNHG6 facilitates cell proliferation, migration, invasion and EMT by upregulating UCK2 and activating the Wnt/beta-catenin signaling in cervical cancer. Bioorg Chem..

[38] Li J., Wang H. (2022). H3K27ac-activated EGFR-AS1 promotes cell growth in cervical cancer through ACTN4-mediated WNT pathway. Biol. Direct.

[39] Song H., Liu Y., Liang H., Jin X., Liu L. (2021). SPINT1-AS1 Drives Cervical Cancer Progression via Repressing miR-214 Biogenesis. Front. Cell Dev. Biol..

[40] Niu Z., Wang F., Lv S., Lv Y., Liu M., Fu L. (2021). HNRNPU-AS1 Regulates Cell Proliferation and Apoptosis via the MicroRNA 205-5p/AXIN2 Axis and Wnt/beta-Catenin Signaling Pathway in Cervical Cancer. Mol. Cell Biol..

[41] Xia L., Chen Y.X., Lian J.B. (2021). LINC00665 promotes HeLa cell proliferation, migration, invasion and epithelial-mesenchymal transition by activating the WNT-CTNNB1/betacatenin signaling pathway. Sheng Li Xue Bao.

[42] Yong J., Groeger S., Meyle J., Ruf S. (2022). MAPK and beta-Catenin signaling: Implication and interplay in orthodontic tooth movement. Front. Biosci..

[43] Zhang P., Zhao F., Jia K., Liu X. (2022). The LOXL1 antisense RNA 1 (LOXL1-AS1)/microRNA-423-5p (miR-423-5p)/ectodermal-neural cortex 1 (ENC1) axis promotes cervical cancer through the mitogen-activated protein kinase (MEK)/extracellular signal-regulated kinase (ERK) pathway. Bioengineered.

[44] Chu D., Liu T., Yao Y., Luan N. (2021). LINC00997/MicroRNA 574-3p/CUL2 Promotes Cervical Cancer Development via Mitogen-Activated Protein Kinase Signaling. Mol. Cell Biol..

[45] Tzavlaki K., Moustakas A. (2020). TGF-beta Signaling. Biomolecules.

[46] Zhang J., Wang Q., Quan Z. (2019). Long non-coding RNA CASC9 enhances breast cancer progression by promoting metastasis through the meditation of miR-215/TWIST2 signaling associated with TGF-beta expression. Biochem. Biophys. Res. Commun..

[47] Cao L., Jin H., Zheng Y., Mao Y., Fu Z., Li X., Dong L. (2019). DANCR-mediated microRNA-665 regulates proliferation and metastasis of cervical cancer through the ERK/SMAD pathway. Cancer Sci..

[48] Wang J., Zhang Y., Lin R., Mao B., Wang W., Bai Y., He W., Liu Q. (2019). Long Noncoding RNA loc285194 Expression in Human Papillomavirus-Positive and -Negative Cervical Squamous Cell Carcinoma, C33A, and SiHa Cells and Transforming Growth Factor-β1. Med. Sci. Monit..

[49] Xu J., Liu X.-Y., Zhang Q., Liu H., Zhang P., Tian Z.-B., Zhang C.-P., Li X.-Y. (2022). Crosstalk Among YAP, LncRNA, and Tumor-Associated Macrophages in Tumorigenesis Development. Front. Oncol..

[50] Zhang Y., Fang Z., Guo X., Dong H., Zhou K., Huang Z., Xiao Z. (2019). lncRNA B4GALT1-AS1 promotes colon cancer cell stemness and migration by recruiting YAP to the nucleus and enhancing YAP transcriptional activity. J. Cell. Physiol..

[51] Li R.-H., Tian T., Ge Q.-W., He X.-Y., Shi C.-Y., Li J.-H., Zhang Z., Liu F.-Z., Sang L.-J., Yang Z.-Z. (2021). A phosphatidic acid-binding lncRNA SNHG9 facilitates LATS1 liquid–liquid phase separation to promote oncogenic YAP signaling. Cell Res..

[52] Lou C., Zhao J., Gu Y., Li Q., Tang S., Wu Y., Tang J., Zhang C., Li Z., Zhang Y. (2019). LINC01559 accelerates pancreatic cancer cell proliferation and migration through YAP-mediated pathway. J. Cell. Physiol..

[53] Li C., Wang S., Xing Z., Lin A., Liang K., Song J. (2017). A ROR1-HER3-lncRNA signalling axis modulates the Hippo-YAP pathway to regulate bone metastasis. Nat. Cell Biol..

[54] Li D., Bao J., Yao J., Li J. (2020). lncRNA USP2-AS1 promotes colon cancer progression by modulating Hippo/YAP1 signaling. Am. J. Transl. Res..

[55] Wang Q., Ding J., Nan G., Lyu Y., Ni G. (2019). LncRNA NOC2L-4.1 functions as a tumor oncogene in cervical cancer progression by regulating the miR-630/YAP1 pathway. J. Cell Biochem..

[56] Zhu H., Zhu C., Feng X., Luo Y. (2021). Long noncoding RNA SNHG3 promotes malignant phenotypes in cervical cancer cells via association with YAP1. Hum. Cell.

[57] Burgess J.T., Rose M., Boucher D., Plowman J., Molloy C., Fisher M., O’Leary C., Richard D.J., O’Byrne K.J., Bolderson E. (2020). The Therapeutic Potential of DNA Damage Repair Pathways and Genomic Stability in Lung Cancer. Front. Oncol..

[58] Tehrani S.S., Karimian A., Parsian H., Majidinia M., Yousefi B. (2018). Multiple Functions of Long Non-Coding RNAs in Oxidative Stress, DNA Damage Response and Cancer Progression. J. Cell Biochem..

[59] Wang X., Liu H., Shi L., Yu X., Gu Y., Sun X. (2018). LINP1 facilitates DNA damage repair through non-homologous end joining (NHEJ) pathway and subsequently decreases the sensitivity of cervical cancer cells to ionizing radiation. Cell Cycle.

[60] Wen D., Huang Z., Li Z., Tang X., Wen X., Liu J., Li M. (2020). LINC02535 co-functions with PCBP2 to regulate DNA damage repair in cervical cancer by stabilizing RRM1 mRNA. J. Cell. Physiol..

[61] Janku F., Yap T.A., Meric-Bernstam F. (2018). Targeting the PI3K pathway in cancer: Are we making headway?. Nat. Rev. Clin. Oncol..

[62] Sanaei M., Khorasani A.B.S., Pourbagheri-Sigaroodi A., Shahrokh S., Zali M.R., Bashash D. (2021). The PI3K/Akt/mTOR axis in colorectal cancer: Oncogenic alterations, non-coding RNAs, therapeutic opportunities, and the emerging role of nanoparticles. J. Cell. Physiol..

[63] Presti D., Quaquarini E. (2019). The PI3K/AKT/mTOR and CDK4/6 Pathways in Endocrine Resistant HR+/HER2- Metastatic Breast Cancer: Biological Mechanisms and New Treatments. Cancers.

[64] Chamcheu J.C., Roy T., Uddin M.B., Banang-Mbeumi S., Chamcheu R.-C.N., Walker A.L., Liu Y.-Y., Huang S. (2019). Role and Therapeutic Targeting of the PI3K/Akt/mTOR Signaling Pathway in Skin Cancer: A Review of Current Status and Future Trends on Natural and Synthetic Agents Therapy. Cells.

[65] Zhu K., Deng C., Du P., Liu T., Piao J., Piao Y., Yang M., Chen L. (2022). G6PC indicated poor prognosis in cervical cancer and promoted cervical carcinogenesis in vitro and in vivo. Reprod. Biol. Endocrinol..

[66] An R., Meng S., Qian H. (2022). Identification of Key Pathways and Establishment of a Seven-Gene Prognostic Signature in Cervical Cancer. J. Oncol..

[67] Jiang L., Jin H., Gong S., Han K., Li Z., Zhang W., Tian J. (2022). LncRNA KCNQ1OT1 -mediated cervical cancer progression by sponging miR -1270 as a ceRNA of LOXL2 through PI3k /Akt pathway. J. Obstet. Gynaecol. Res..

[68] Liu H., Zhang L., Ding X., Sui X. (2020). LINC00861 inhibits the progression of cervical cancer cells by functioning as a ceRNA for miR-513b-5p and regulating the PTEN/AKT/mTOR signaling pathway. Mol. Med. Rep..

[69] Shi W.-J., Liu H., Ge Y.-F., Wu D., Tan Y.-J., Shen Y.-C., Wang H., Xu H. (2020). LINC00673 exerts oncogenic function in cervical cancer by negatively regulating miR-126-5p expression and activates PTEN/PI3K/AKT signaling pathway. Cytokine.

[70] Vito A., El-Sayes N., Mossman K. (2020). Hypoxia-Driven Immune Escape in the Tumor Microenvironment. Cells.

[71] Shen Y., Liu Y., Sun T., Yang W. (2017). LincRNA-p21 knockdown enhances radiosensitivity of hypoxic tumor cells by reducing autophagy through HIF-1/Akt/mTOR/P70S6K pathway. Exp. Cell Res..

[72] Yang J., Yang M., Lv H., Zhou M., Mao X., Qin X., Xu Y., Li L., Xing H. (2022). lncRNA SNHG15 Induced by SOX12 Promotes the Tumorigenic Properties and Chemoresistance in Cervical Cancer via the miR-4735-3p/HIF1a Pathway. Oxidative Med. Cell. Longev..

[73] Li L., Ma Y., Maerkeya K., Reyanguly D., Han L. (2021). LncRNA OIP5-AS1 Regulates the Warburg Effect Through miR-124-5p/IDH2/HIF-1alpha Pathway in Cervical Cancer. Front Cell Dev. Biol..

[74] Li X., Zhang C., Tian Y. (2021). Long non-coding RNA TDRG1 promotes hypoxia-induced glycolysis by targeting the miR-214-5p/SEMA4C axis in cervical cancer cells. Histochem. J..

[75] Ta W., Zhang Y., Zhang S., Sun P. (2019). LncRNA ANCR downregulates hypoxia-inducible factor 1α and inhibits the growth of HPV-negative cervical squamous cell carcinoma under hypoxic conditions. Mol. Med. Rep..

[76] Ozaki T., Nakagawara A. (2011). Role of p53 in Cell Death and Human Cancers. Cancers.

[77] Harris S.L., Levine A.J. (2005). The p53 pathway: Positive and negative feedback loops. Oncogene.

[78] Abdel-Latif M., Riad A., Soliman R.A., Elkhouly A.M., Nafae H., Gad M.Z. (2022). MALAT-1/p53/miR-155/miR-146a ceRNA circuit tuned by methoxylated quercitin glycoside alters immunogenic and oncogenic profiles of breast cancer. Mol. Cell Biochem..

[79] Ghafouri-Fard S., Sohrabi B., Hussen B.M., Mehravaran E., Jamali E., Arsang-Jang S., Fathi M., Taheri M., Samsami M. (2022). Down-regulation of MEG3, PANDA and CASC2 as p53-related lncRNAs in breast cancer. Breast Dis..

[80] Xu M., Zhao X., Zhao S., Yang Z., Yuan W., Han H. (2021). Landscape analysis of lncRNAs shows that DDX11-AS1 promotes cell-cycle progression in liver cancer through the PARP1/p53 axis. Cancer Lett..

[81] Pal S., Garg M., Pandey A.K. (2020). Deciphering the Mounting Complexity of the p53 Regulatory Network in Correlation to Long Non-Coding RNAs (lncRNAs) in Ovarian Cancer. Cells.

[82] Zhao Y., Li Y., Sheng J., Wu F., Li K., Huang R., Wang X., Jiao T., Guan X., Lu Y. (2019). P53-R273H mutation enhances colorectal cancer stemness through regulating specific lncRNAs. J. Exp. Clin. Cancer Res..

[83] Li X.L., Subramanian M., Jones M.F., Chaudhary R., Singh D.K., Zong X., Gryder B., Sindri S., Mo M., Schetter A. (2017). Long Noncoding RNA PURPL Suppresses Basal p53 Levels and Promotes Tumorigenicity in Colorectal Cancer. Cell Rep..

[84] Chaudhary R., Gryder B., Woods W.S., Subramanian M., Jones M.F., Li X.L., Jenkins L.M., Shabalina S.A., Mo M., Dasso M. (2017). Prosurvival long noncoding RNA PINCR regulates a subset of p53 targets in human colorectal cancer cells by binding to Matrin 3. eLife.

[85] Zhu Y., Ren C., Jiang D., Yang L., Chen Y., Li F. (2021). RPL34-AS1-induced RPL34 inhibits cervical cancer cell tumorigenesis via the MDM2-P53 pathway. Cancer Sci..

[86] Sharma S., Munger K. (2020). Expression of the Long Noncoding RNA DINO in Human Papillomavirus-Positive Cervical Cancer Cells Reactivates the Dormant TP53 Tumor Suppressor through ATM/CHK2 Signaling. mBio.

[87] Cui L., Nai M., Zhang K., Li L., Li R. (2019). lncRNA WT1-AS inhibits the aggressiveness of cervical cancer cell via regulating p53 expression via sponging miR-330-5p. Cancer Manag. Res..

[88] Meza-Sosa K.F., Miao R., Navarro F., Zhang Z., Zhang Y., Hu J.J., Hartford C.C.R., Li X.L., Pedraza-Alva G., Pérez-Martínez L. (2022). SPARCLE, a p53-induced lncRNA, controls apoptosis after genotoxic stress by promoting PARP-1 cleavage. Mol. Cell.

[89] Sun Y.F., Wang Y., Li X.D., Wang H. (2022). SNHG15, a p53-regulated lncRNA, suppresses cisplatin-induced apoptosis and ROS accumulation through the miR-335-3p/ZNF32 axis. Am. J. Cancer Res..

[90] Ou X., Zhou X., Li J., Ye J., Liu H., Fang D., Cai Q., Cai S., He Y., Xu J. (2022). p53-Induced LINC00893 Regulates RBFOX2 Stability to Suppress Gastric Cancer Progression. Front. Cell Dev. Biol..

[91] Wang C., Yang Y., Wu X., Li J., Liu K., Fang D., Li B., Shan G., Mei X., Wang F. (2022). Reciprocal modulation of long noncoding RNA EMS and p53 regulates tumorigenesis. Proc. Natl. Acad. Sci. USA.

[92] Rong H., Chen B., Ma K., Wei X., Peng J., Zhu J. (2021). Downregulation of lncRNA LINC-PINT Participates in the Recurrence of Esophageal Squamous Cell Carcinoma Possibly by Interacting miRNA-21. Cancer Biotherapy Radiopharm..

[93] Jin S., Yang X., Li J., Yang W., Ma H., Zhang Z. (2019). p53-targeted lincRNA-p21 acts as a tumor suppressor by inhibiting JAK2/STAT3 signaling pathways in head and neck squamous cell carcinoma. Mol. Cancer.

[94] Zhang E.-B., Yin D.-D., Sun M., Kong R., Liu X.-H., You L.-H., Han L., Xia R., Wang K.-M., Yang J.-S. (2014). P53-regulated long non-coding RNA TUG1 affects cell proliferation in human non-small cell lung cancer, partly through epigenetically regulating HOXB7 expression. Cell Death Dis..

[95] Mello S.S., Sinow C., Raj N., Mazur P.K., Bieging-Rolett K., Broz D.K., Imam J.F.C., Vogel H., Wood L.D., Sage J. (2017). Neat1 is a p53-inducible lincRNA essential for transformation suppression. Genes Dev..

[96] Schmitt A., Garcia J.T., Hung T., Flynn R., Shen Y., Qu K., Payumo A.Y., Peres-Da-Silva A., Broz D.K., Baum R. (2016). An inducible long noncoding RNA amplifies DNA damage signaling. Nat. Genet..

[97] Zhu H., Chen X., Hua Z., Shi Z., Zhou Q., Zhengzheng S., Wang Y. (2017). Long non-coding RNA expression profile in cervical cancer tissues. Oncol. Lett..

[98] Wang H., Zhao Y., Chen M., Cui J. (2017). Identification of Novel Long Non-coding and Circular RNAs in Human Papillomavirus-Mediated Cervical Cancer. Front. Microbiol..

[99] Olgun G., Sahin O., Tastan O. (2018). Discovering lncRNA mediated sponge interactions in breast cancer molecular subtypes. BMC Genom..

[100] Su Y., Hou W., Zhang C., Ji P., Hu R., Zhang Q., Wang Y., Li P., Zhang H., Chen Y. (2022). Long non-coding RNA ZFAS1 regulates cell proliferation and invasion in cervical cancer via the miR-190a-3p/KLF6 axis. Bioengineered.

[101] Liu Y., Chen J., Zhou L., Yin C. (2022). LINC00885 promotes cervical cancer progression through sponging miR-3150b-3p and upregulating BAZ2A. Biol. Direct.

[102] Ji F., Lu Y., Chen S., Lin X., Yu Y., Zhu Y., Luo X. (2021). m6A methyltransferase METTL3-mediated lncRNA FOXD2-AS1 promotes the tumorigenesis of cervical cancer. Mol. Ther.-Oncolytics.

[103] Lv H., Jin S., Zou B., Liang Y., Xie J., Wu S. (2021). Analyzing the whole-transcriptome profiles of ncRNAs and predicting the competing endogenous RNA networks in cervical cancer cell lines with cisplatin resistance. Cancer Cell Int..

[104] Xia M., Duan L.J., Lu B.N., Pang Y.Z., Pang Z.R. (2021). LncRNA AFAP1-AS1/miR-27b-3p/VEGF-C axis modulates stemness characteristics in cervical cancer cells. Chin. Med. J..

[105] Zhang J., Liu H.-L., Liu J.-B., Zhang Y., Li Y.-H. (2021). LncRNA AL592284.1 facilitates proliferation and metastasis of cervical cancer cells via miR-30a-5p/Vimentin/EMT axis. Biochem. Biophys. Res. Commun..

[106] Yang W., Wang X., Song S., Chu Y., Sun D., Yu X., Zou Y. (2021). Long noncoding RNA ALOX12-AS1 inhibits cervical cancer cells proliferation via targeting miR-3171. Anti-Cancer Drugs.

[107] He S., Feng Y., Zou W., Wang J., Li G., Xiong W., Xie Y., Ma J., Liu X. (2021). The Role of the SOX9/lncRNA ANXA2P2/miR-361-3p/SOX9 Regulatory Loop in Cervical Cancer Cell Growth and Resistance to Cisplatin. Front Oncol..

[108] Gao S., Lv Q., Xu F., Li H., Guo X. (2021). LncRNA CASC9-1 Facilitates Cell Malignant Behaviors in Cervical Squamous Cell Carcinoma by Targeting miR-383-5p to Up-regulate MAPKAP1. Arch. Med. Res..

[109] Wang J., Liu Y., Cai H., Jiang H., Li W., Shi Y. (2021). Long coding RNA CCAT2 enhances the proliferation and epithelial-mesenchymal transition of cervical carcinoma cells via the microRNA-493-5p/CREB1 axis. Bioengineered.

[110] Hu C., Han Y., Zhu G., Li G., Wu X. (2021). Krüppel-like factor 5-induced overexpression of long non-coding RNA DANCR promotes the progression of cervical cancer via repressing microRNA-145-3p to target ZEB1. Cell Cycle.

[111] Kong X., Wang J.S., Yang H. (2021). Upregulation of lncRNA DARS-AS1 accelerates tumor malignancy in cervical cancer by activating cGMP-PKG pathway. J. Biochem Mol. Toxicol..

[112] He M., Wang Y., Cai J., Xie Y., Tao C., Jiang Y., Li H., Song F. (2021). LncRNA DLEU2 promotes cervical cancer cell proliferation by regulating cell cycle and NOTCH pathway. Exp. Cell Res..

[113] Gu J., Liu Y., Qi T., Qian W., Hu D., Feng W. (2021). Long non-coding RNA DUXAP8 elevates RCN2 expression and facilitates cell malignant behaviors and angiogenesis in cervical cancer via sponging miR-1297. Diagn. Pathol..

[114] Huang X., Shi H., Shi X., Jiang X. (2021). LncRNA FBXL19-AS1 promotes proliferation and metastasis of cervical cancer through upregulating COL1A1 as a sponge of miR-193a-5p. J. Biol. Res..

[115] Yang X., Qu Y., Zhang J. (2021). Up-Regulated LncRNA FEZF1-AS1 Promotes the Progression of Cervical Carcinoma Cells via MiR-367-3p/SLC12A5 Signal Axis. Arch. Med. Res..

[116] Liu G., Du X., Xiao L., Zeng Q., Liu Q. (2021). Activation of FGD5-AS1 Promotes Progression of Cervical Cancer through Regulating BST2 to Inhibit Macrophage M1 Polarization. J. Immunol. Res..

[117] Yang X., Du H., Bian W., Li Q., Sun H. (2021). FOXD3AS1/miR1283p/LIMK1 axis regulates cervical cancer progression. Oncol. Rep..

[118] Gao Y., Zou T., Liang W., Zhang Z., Qie M. (2021). Long non-coding RNA HAND2-AS1 delays cervical cancer progression via its regulation on the microRNA-21-5p/TIMP3/VEGFA axis. Cancer Gene Ther..

[119] Trujano-Camacho S., Cantu-de Leon D., Delgado-Waldo I., Coronel-Hernandez J., Millan-Catalan O., Hernandez-Sotelo D., Lopez-Camarillo C., Perez-Plasencia C., Campos-Parra A.D. (2021). Inhibition of Wnt-beta-Catenin Signaling by ICRT14 Drug Depends of Post-Transcriptional Regulation by HOTAIR in Human Cervical Cancer HeLa Cells. Front. Oncol..

[120] Zhang W., Wu Q., Liu Y., Wang X., Ma C., Zhu W. (2021). LncRNA HOTAIR Promotes Chemoresistance by Facilitating Epithelial to Mesenchymal Transition through miR-29b/PTEN/PI3K Signaling in Cervical Cancer. Cells Tissues Organs.

[121] Zhang W., Liu J., Wu Q., Liu Y., Ma C. (2021). HOTAIR Contributes to Stemness Acquisition of Cervical Cancer through Regulating miR-203 Interaction with ZEB1 on Epithelial-Mesenchymal Transition. J. Oncol..

[122] Zhou Y., Wang Y., Lin M., Wu D., Zhao M. (2021). LncRNA HOTAIR promotes proliferation and inhibits apoptosis by sponging miR-214-3p in HPV16 positive cervical cancer cells. Cancer Cell Int..

[123] Chen R., He P. (2021). Long noncoding RNA HOXA-AS2 accelerates cervical cancer by the miR-509-3p/BTN3A1 axis. J. Pharm. Pharmacol..

[124] Wang T., Li W., Ye B., Zhang S., Lei X., Zhang D. (2021). FTO-stabilized lncRNA HOXC13-AS epigenetically upregulated FZD6 and activated Wnt/beta-catenin signaling to drive cervical cancer proliferation, invasion, and EMT. J. BUON..

[125] Zhao R., Song J., Jin Y., Liu Y. (2021). Long noncoding RNA HOXC-AS3 enhances the progression of cervical cancer via activating ErbB signaling pathway. Histochem. J..

[126] Liu H., Liu L., Liu Q., He F., Zhu H. (2022). LncRNA HOXD-AS1 affects proliferation and apoptosis of cervical cancer cells by promoting FRRS1 expression via transcription factor ELF1. Cell Cycle.

[127] Zhu L., Chen R., Jiang C., Xie Q., Zhao W., Gao X. (2021). Mechanism underlying long noncoding RNA ILF3AS1mediated inhibition of cervical cancer cell proliferation, invasion and migration, and promotion of apoptosis. Mol. Med. Rep..

[128] Liu J., Wang Y. (2021). Long non-coding RNA KCNQ1OT1 facilitates the progression of cervical cancer and tumor growth through modulating miR-296-5p/HYOU1 axis. Bioengineered.

[129] Tian L., Han F., Yang J., Ming X., Chen L. (2021). Long non-coding RNA LINC01006 exhibits oncogenic properties in cervical cancer by functioning as a molecular sponge for microRNA-28-5p and increasing PAK2 expression. Int. J. Mol. Med..

[130] Zhai Y., Liu Y., Wang Z., Wang W., Zhou J., Lu J. (2021). Long Non-Coding RNA LINC00313 Accelerates Cervical Carcinoma Progression by miR-4677-3p/CDK6 Axis. OncoTargets Ther..

[131] Liu X., Shen X., Zhang J. (2021). Long non-coding RNA LINC00514 promotes the proliferation and invasion through the miR -708-5p/ HOXB3 axis in cervical squamous cell carcinoma. Environ. Toxicol..

[132] Liu Y., Qiu S., Zheng X., Qiu Y., Yao S., Ge Y. (2021). LINC00662 modulates cervical cancer cell proliferation, invasion, and apoptosis via sponging miR-103a-3p and upregulating PDK4. Mol. Carcinog..

[133] Huang S.-K., Ni R.-X., Wang W.-J., Wang D., Zhao M., Lei C.-Z., Sun X.-J., Huang C.-Z., Bai P., Che Y.-Q. (2021). Overexpression of LINC00673 Promotes the Proliferation of Cervical Cancer Cells. Front. Oncol..

[134] Guo H., Li J., Fan F., Zhou P. (2021). LINC00707 Regulates miR-382-5p/VEGFA Pathway to Enhance Cervical Cancer Progression. J. Immunol. Res..

[135] Liu Y., Tu H., Zhang L., Xiong J., Li L. (2021). FOXP3-induced LINC00885 promotes the proliferation and invasion of cervical cancer cells. Mol. Med. Rep..

[136] Chen Y., Gu Y., Gu Y., Wu J. (2021). Long Noncoding RNA LINC00899/miR-944/ESR1 Axis Regulates Cervical Cancer Cell Proliferation, Migration, and Invasion. J. Interferon Cytokine Res..

[137] Zhang D., Zhang Y., Sun X. (2020). LINC01133 promotes the progression of cervical cancer via regulating miR-30a-5p/FOXD1. Asia-Pacific J. Clin. Oncol..

[138] Liao Y., Huang J., Liu P., Zhang C., Liu J., Xia M., Shang C., Ooi S., Chen Y., Qin S. (2022). Downregulation of LNMAS orchestrates partial EMT and immune escape from macrophage phagocytosis to promote lymph node metastasis of cervical cancer. Oncogene.

[139] Chai Y., Wang L., Qu Y., Hu Z. (2022). LncRNA MAGI2-As3 Suppresses the Proliferation and Invasion of Cervical Cancer by Sponging MiR-15b. J. Heal. Eng..

[140] Wang T., Zhang W., Huang W., Hua Z., Li S. (2021). LncRNA MALAT1 was regulated by HPV16 E7 independently of pRB in cervical cancer cells. J. Cancer.

[141] Tie W., Ge F. (2021). MALAT1 Inhibits Proliferation of HPV16-Positive Cervical Cancer by Sponging miR-485-5p to Promote Expression of MAT2A. DNA Cell Biol..

[142] Liang T., Wang Y., Jiao Y., Cong S., Jiang X., Dong L., Zhang G., Xiao D. (2021). LncRNA MALAT1 Accelerates Cervical Carcinoma Proliferation by Suppressing miR-124 Expression in Cervical Tumor Cells. J. Oncol..

[143] Wang X., Zhang C., Gong M., Jiang C. (2021). A Novel Identified Long Non-coding RNA, lncRNA MEF2C-AS1, Inhibits Cervical Cancer via Regulation of miR-592/RSPO1. Front. Mol. Biosci..

[144] Pan X., Cao Y.-M., Liu J.-H., Ding J., Xie X.-Y., Cao P.-G. (2021). MEG3 Induces Cervical Carcinoma Cells’ Apoptosis Through Endoplasmic Reticulum Stress by miR-7-5p/STC1 Axis. Cancer Biotherapy Radiopharm..

[145] Hu Y.L., Zhang Y.X., Liu N., Liu H., Yuan Y.C. (2021). LncRNA MIR503HG regulated cell viability, metastasis and apoptosis of cervical cancer via miR-191/CEBPB axis. Eur. Rev. Med. Pharmacol. Sci..

[146] Geng F., Jia W.C., Li T., Li N., Wei W. (2022). Knockdown of lncRNA NEAT1 suppresses proliferation and migration, and induces apoptosis of cervical cancer cells by regulating the miR377/FGFR1 axis. Mol. Med. Rep..

[147] Shao X., Zheng X., Ma D., Liu Y., Liu G. (2021). Inhibition of lncRNA-NEAT1 sensitizes 5-Fu resistant cervical cancer cells through de-repressing the microRNA-34a/LDHA axis. Biosci. Rep..

[148] Zhang L., Cai Y., Tian C., Li Y., Ma K., Gao X. (2022). LncRNA Opa interacting protein 5-antisense RNA 1 (OIP5-AS1) promotes the migration, invasion and epithelial-mesenchymal transition (EMT) through targeting miR-147a/insulin-like growth factor 1 receptor (IGF1R) pathway in cervical cancer tissues and cell model. J. Obstet. Gynaecol. Res..

[149] Hou H., Yu R., Zhao H., Yang H., Hu Y., Hu Y., Guo J. (2021). LncRNA OTUD6B-AS1 Induces Cisplatin Resistance in Cervical Cancer Cells Through Up-Regulating Cyclin D2 via miR-206. Front. Oncol..

[150] Cao S., Li H., Li L. (2021). LncRNA SNHG17 Contributes to the Progression of Cervical Cancer by Targeting microRNA-375-3p. Cancer Manag. Res..

[151] Zhang L., Wu X., Li Y., Teng X., Zou L., Yu B. (2021). LncRNA SNHG5 promotes cervical cancer progression by regulating the miR-132/SOX4 pathway. Autoimmunity.

[152] An M., Xing X., Chen T. (2021). Long non-coding RNA UCA1 enhances cervical cancer cell proliferation and invasion by regulating microRNA-299-3p expression. Oncol. Lett..

[153] Fu J., Zhang Y., Wang M., Hu J., Fang Y. (2021). Inhibition of the long non-coding RNA UNC5B-AS1/miR-4455/RSPO4 axis reduces cervical cancer growth in vitro and in vivo. J. Gene Med..

[154] Chen M., Chi Y., Chen H., Zhao L. (2021). Long non-coding RNA USP30-AS1 aggravates the malignant progression of cervical cancer by sequestering microRNA-299-3p and thereby overexpressing PTP4A1. Oncol. Lett..

[155] Tong W., Zhang H. (2021). Overexpression of long non-coding RNA WT1-AS or silencing of PIK3AP1 are inhibitory to cervical cancer progression. Cell Cycle.

[156] Luo H., Zhang J., He Z., Wu S. (2021). Long Noncoding RNA WT1-AS Inhibits the Progression of Cervical Cancer by Sponging miR-205. Cancer Biotherapy Radiopharm..

[157] Hsu P., Shi H., He C. (2017). Epitranscriptomic influences on development and disease. Genome Biol..

[158] Rauch S., He C., Dickinson B.C. (2018). Targeted m6A Reader Proteins to Study Epitranscriptomic Regulation of Single RNAs. J. Am. Chem. Soc..

[159] Jia G., Fu Y., He C. (2012). Reversible RNA adenosine methylation in biological regulation. Trends Genet..

[160] Motorin Y., Helm M. (2021). RNA nucleotide methylation: 2021 update. Wiley Interdiscip. Rev. RNA.

[161] Yin L., Zhu X., Novák P., Zhou L., Gao L., Yang M., Zhao G., Yin K. (2021). The epitranscriptome of long noncoding RNAs in metabolic diseases. Clin. Chim. Acta.

[162] Zhang H., Kong W., Zhao X., Han C., Liu T., Li J., Song D. (2022). N6-Methyladenosine-Related lncRNAs as potential biomarkers for predicting prognoses and immune responses in patients with cervical cancer. BMC Genom. Data.

[163] Zhang Y., Wang D., Wu D., Zhang D., Sun M. (2020). Long Noncoding RNA KCNMB2-AS1 Stabilized by N6-Methyladenosine Modification Promotes Cervical Cancer Growth Through Acting as a Competing Endogenous RNA. Cell Transplant..

[164] Wang X., Zhang J., Wang Y. (2019). Long noncoding RNA GAS5-AS1 suppresses growth and metastasis of cervical cancer by increasing GAS5 stability. Am. J. Transl. Res..

